# Evaluating the Role of Vitamin D in Prediabetes Management, Insights from RCTs in the MENA Region: A Comprehensive Systematic Review

**DOI:** 10.3390/jcm14041239

**Published:** 2025-02-13

**Authors:** Mohammed A. M. Y. Al-Hetar, Noradliyanti Rusli, Mohd Amir Kamaruzzaman, Husni Al-Goshae, Wan Zurinah Wan Ngah, Shamsul Azhar Shah, Abdullah Mohammed Al-Matary, Qais Mohammed Al-Hetar, Dhya’a Alhaq Mohammed Senan, Norasyikin A. Wahab

**Affiliations:** 1 Department of Medicine, Faculty of Medicine, Universiti Kebangsaan Malaysia, Jalan Yaacob Latif, Cheras, Kuala Lumpur 56000, Malaysia; p143260@siswa.ukm.edu.my (M.A.M.Y.A.-H.); p116652@siswa.ukm.edu.my (N.R.); 2 Medical City Complex, The Specialized Clinic for Endocrinology and Diabetes, Ibb 6001196, Yemen; qaismalhetar@gmail.com (Q.M.A.-H.); dhyaaalhaqsenan@gmail.com (D.A.M.S.); 3 Department of Anatomy, Faculty of Medicine, Universiti Kebangsaan Malaysia, Jalan Yaacob Latif, Cheras, Kuala Lumpur 56000, Malaysia; mohdamir@ukm.edu.my; 4 Department of Anatomy, International Medical School, Management & Science University, Shah Alam 40100, Selangor, Malaysia; husni_ahmed@msu.edu.my; 5 Department of Biochemistry, Faculty of Medicine, Universiti Kebangsaan Malaysia, Jalan Yaacob Latif, Cheras, Kuala Lumpur 56000, Malaysia; wwanzurinah@yahoo.com; 6 Medical Innovation Research Centre, Shiga University of Medical Sciences, Otsu 520-2192, Shiga, Japan; 7 Department of Community Health, Faculty of Medicine, Universiti Kebangsaan Malaysia, Jalan Yaacob Latif, Cheras, Kuala Lumpur 56000, Malaysia; drsham@hctm.ukm.edu.my; 8 Department of Surgery, Jiblah University for Medical and Health Sciences, Ibb 6001196, Yemen; almatari95@gmail.com; 9 Hospital Canselor Tuanku Muhriz, Jalan Yaacob Latif, Bandar Tun Razak, Cheras, Kuala Lumpur 56000, Malaysia

**Keywords:** vitamin D, 25 hydroxy vitamin D, prediabetes, insulin resistance, MENA region, RCTs

## Abstract

**Background/Objectives**: The association between vitamin D deficiency and prediabetes has been extensively investigated, yet the findings remain inconsistent, with limited data available on the MENA region. This systematic review aims to assess the relationship between vitamin D deficiency and prediabetes in the Middle East and North Africa (MENA) region, focusing specifically on randomized controlled trials (RCTs). **Methods**: A comprehensive literature search was performed across four databases, which were Ovid MEDLINE, Cochrane, Scopus, and PubMed. RCTs studies conducted on people with prediabetes aged 15 years and older who live in the MENA region, and receiving vitamin D supplementation were included in the study. **Results**: From 2194 studies identified from the literature search, only 51 studies were considered eligible for full-text review. Ultimately, seven articles were finalized for inclusion. The findings from these studies showed mixed results, where some studies indicated that vitamin D supplementation had no significant effect on these outcomes. The remaining reported improvements in insulin sensitivity and a reduced risk of progression to type 2 diabetes with vitamin D supplementation. **Conclusions**: This systematic review examines the complex and contradictory relationship between vitamin D deficiency and prediabetes in the MENA region. Due to the mixed pattern seen in the intervention of vitamin D to prevent the development of type 2 diabetes, further research is necessary to elucidate the underlying mechanisms and potential confounding factors specifically in population of the MENA region.

## 1. Introduction

**Figure 1:**
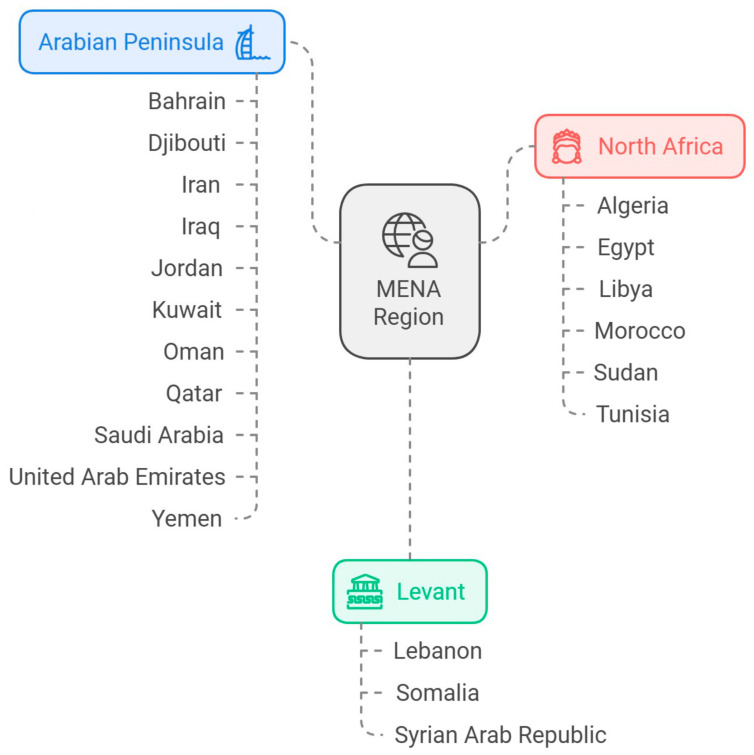
The historical culture of the MENA region as well as its recent developments very much influence the health care sector, which is a mixture of traditional and adoptive modern technology. The region is one of the most naturally blessed and populous regions; thus, it comes with increased social issues like environmental health issues, non-communicable diseases, and health inequalities, occasioned by the new increase in urbanization and the social-economic divide. Nevertheless, there is tactical relevance since the region has a chance to encounter common health problems, the development of the health care system, and the management of epidemics. Various cultural, economic, and geopolitical forces, for instance, bear influence on crafting relevant region-specific health policies and programs.

Vitamin D, or calciferol, is a fat-soluble vitamin which normally can be found in some foods, including fatty fish, liver, red meat, egg yolks, fortified dairy, and grain products. Apart from food, vitamin D can also be synthesized in our skin upon exposure to ultraviolet (UV) radiation from sunshine [1]. However, vitamin D insufficiency is increasing worldwide and becoming a significant public health issue in the Middle East, with prevalence rates varying across different populations and regions, especially in the Middle East and North Africa (MENA) countries as shown in Figure 1. Despite having higher levels of sun exposure, studies indicate that the MENA region has elevated rates of vitamin D insufficiency that can range from 40% to as high as 90% in certain demographics, particularly among women and children. In 2017, about 90% of the UAE experienced this shortage, and this trend continues to persist [2]. A cross-sectional study indicates that vitamin D insufficiency is notably frequent among females and the younger demographic in the Kurdistan area of Iraq [3]. Moreover, the incidence of vitamin D insufficiency among women was found to be significant in Riyadh, Saudi Arabia [4]. Other studies in Bahrain revealed that vitamin D deficiency was observed in 78.3% of children, which is closely related to the rates in neighboring countries such as Kuwait and Oman, where vitamin D deficiency is similarly high [5]. Recent cross-sectional studies conducted in Iran identified vitamin D deficiency in a range of 25–85% across different age types [6,7].

Multiple elements help explain why many people in the Middle East lack vitamin D. Despite receiving plenty of sunlight, most people in the Middle East still do not get enough direct sun exposure. Traditional cultural dress codes that cover most body parts reduce women’s time in the sun [8]. Both poor vitamin D intake in the diet and the absence of food fortification programs contribute to higher levels of vitamin D deficiency in affected populations [9]. Research shows that 81.1% of Iranian women who seek hospital care at university facilities have vitamin D deficiencies caused by inadequate diet and restricted sun exposure [10]. Evidence shows vitamin D levels decrease during winter in many countries and in Gulf states when people spend more time indoors to escape summer heat, which reduces their vitamin D intake [11]. Winter months in Gulf states and most countries make it harder for health officials to control vitamin D deficiency. Low vitamin D levels harm our health by increasing our risk for many diseases like bone problems, heart diseases, and diabetes [12].

**Figure 2:**
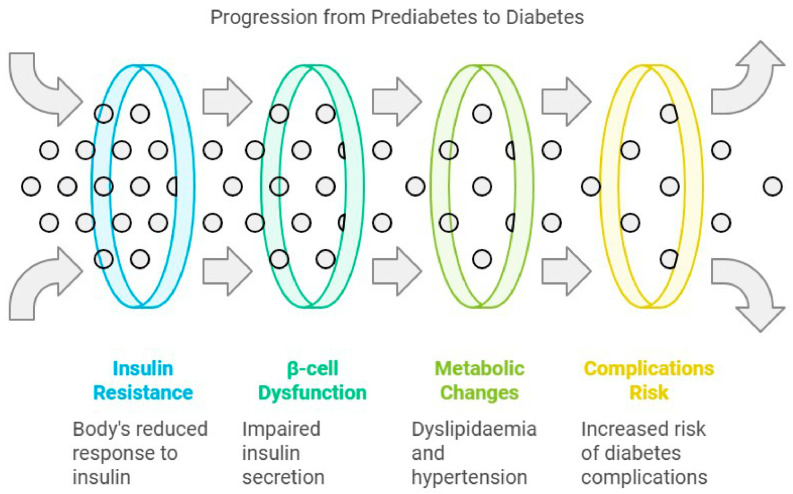
The evolution through prediabetes to diabetes; the stages of the disease are also indicated. A breakdown of the key stages is as follows: Diabetes is the condition that results from high blood sugar due to the inability of the body to use insulin properly. Failure in insulin secretion elevates the blood glucose level, leading to higher stages of diabetes. These metabolic changes increase the chances of cardiovascular disorders and other complications related to diabetes. Constant high blood sugar levels cause damage in all the organs and systems, and it is even worse since the damage is permanent.

**Figure 3:**
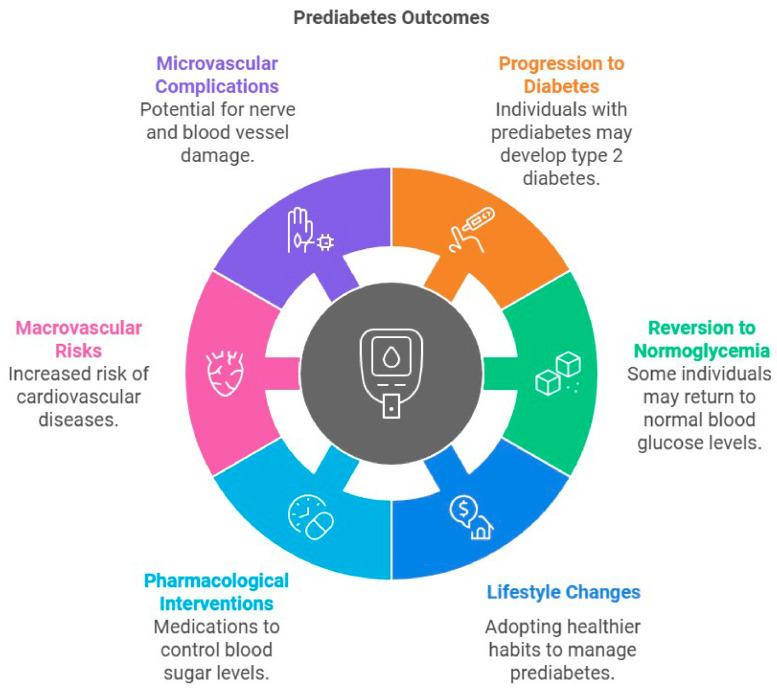
This diagram illustrates six potential pathways for the evolution of prediabetes. Badly managed, prediabetes can progress to earlier-developing diabetes and other complications such as microvascular issues, where nerves and blood vessels are damaged, and macrovascular issues, where the odds of cardiovascular disease are elevated. Nonetheless, there are some patients who can revert back to normal glycaemia just by modifying their diet and increasing their activity levels, or diabetic patients via drug treatment such as with antidiabetic agents and oral hypoglycemic agents. These outcomes underline the need of intervention to reduce hazards and optimize health in related future periods.

Prediabetes is a condition marked by blood glucose levels that are increased beyond the normal range yet fall short of the threshold for diabetes. It is characterized by impaired fasting glucose and/or glucose tolerance, representing an initial phase that may progress to type 2 diabetes [13,14]. The fundamental process involves insulin resistance and β-cell dysfunction, wherein insulin impairs insulin production, leading to fast oscillations and diminished amplitude of substantial insulin pulses [15]. Additional contributing aspects encompass heightened lipolysis, inflammation, and diminished incretin responsiveness [13,15]. This substantially influences metabolic condition, as patients with prediabetes encounter an increased likelihood of advancing to diabetes and its associated problems [16] (Figure 2). Prediabetes is identified as a detrimental metabolic condition that may result in both microvascular and macrovascular problems often linked to diabetes [15]. Microvascular complications such as diabetic peripheral neuropathy begin even during prediabetes stages due to the increase in oxidative stress and inflammatory markers [17]. It is of increasing concern that, each year, 5–10% of individuals with prediabetes progress to diabetes, while a similar proportion may revert to normoglycemia [18]. Excessive fat as an energy source is a prevalent metabolic trait, with obesity being the principal acquired factor that hinders insulin function [19]. The prediabetic condition is also associated with dyslipidemia and increased blood pressure [19]. Effective management, which includes lifestyle changes and pharmacological interventions, is crucial to prevent the progression to type 2 diabetes and mitigate macrovascular risks [15] (Figure 3). However, preventive treatment is still insufficient, while lifestyle changes often fail to become established due to poor compliance.

Vitamin D plays a crucial role not only in bone strength, but also in influencing pancreatic insulin release and sensitivity. In terms of bone health, it acts as a mediator of metabolism and a regulator of inflammation [20]. According to the systematic review by Agata Pienkowska et al., results from seven studies indicated that taking vitamin D supplements does not decrease insulin resistance or decrease the risk of progressing to type 2 diabetes in individuals with prediabetes [21]. Only one study reported improvements in fasting glucose levels and HOMA-IR [21]. The rest of the studies found no effect of high-dose vitamin D supplement in prediabetic patients on glycemic indices, blood pressure, or lipid status [22]. Although some evidence suggests that vitamin D may improve insulin sensitivity and reduce the risk of diabetes, the overall findings are inconsistent [23].

In the quest to uncover the secrets of vitamin D, we recognize the need for more trials and precise investigations. Personalized investigations will help reveal the truth for individuals suffering from vitamin D deficiencies or unique sensitivities. Therefore, we continue our pursuit of clarity under the sun’s golden gaze [24]. Rationale: Considering the elevated prevalence of vitamin D insufficiency and prediabetes in the MENA region, it is essential to investigate the potential correlation between these two conditions. The diverse results from prior studies underscore the necessity of meticulously examining existing research [20,25,26]. A thorough evaluation is required to ascertain the efficacy of vitamin D supplementation in the management of prediabetes and the prevention of type 2 diabetes onset. Given that the accessibility for certain individuals in the MENA region remains below the target for effective management of prediabetes and diabetes per guidelines, an alternative strategy involves exposure to vitamin D sources, either through sunlight or supplementation.

Thus, the aim of this systematic review of randomized controlled trials (RCTs) is to synthesize evidence on the impact of vitamin D supplementation on insulin sensitivity and glycemic management, specifically in people with prediabetes. Additionally, we intend to assess the potential mechanism by which vitamin D affects glucose metabolism and the progression to type 2 diabetes in people with prediabetes. Based on the findings of this systematic review, we will also provide recommendations for future research and public health interventions.

## 2. Materials and Methods

A systematic review of randomized controlled trials, published until August 2024, was conducted following the Preferred Reporting Items for Systematic Reviews and Meta-Analyses (PRISMA) guidelines [27]. The research procedure was initiated by formulating the following clinical question: What is the effect of vitamin D supplementation on individuals with prediabetes in the MENA region? Additionally, the protocol for this systematic review was registered in the international prospective register of systematic reviews, PROSPERO (https://www.crd.york.ac.uk/prospero/ (accessed on 15 October 2024)), to ensure the transparency and quality of this systematic review. The registration number is CRD42024597538.

### 2.1. Search Strategies

An extensive literature search was performed across four databases, namely, Ovid MEDLINE, Cochrane, Scopus, and PubMed, to identify the potential studies that explore the relationship between vitamin D deficiency and prediabetes in the MENA region. We searched for randomized controlled trials using vitamin D supplementation in the MENA region and published in English until August 2024 using the search strategy shown in Table 1. The search strategy included a combination of keywords and Medical Subject Headings (MeSH) terms related to vitamin D deficiency, prediabetes, and the MENA region. Prespecified search engines for each database were used during the search after careful consideration and discussion among MAMYAH, NR, MAK, and NAW, and the details are provided in the Supplementary Materials (S1). The search was performed from 9 to 12 September 2024, and all the data were pooled and kept in EndNote (Version 20) software (Clarivate, London, UK). All duplicates were removed from the pooled data using similar software, and the remaining articles were then transferred into Microsoft Excel for screening.

### 2.2. Selection Criteria and Eligibility Criteria

The Population, Intervention, Comparison, and Outcomes (PICO) model served as the foundation for the selection criteria used to determine the inclusion criteria for the studies. There were five primary components of identification: prediabetes and vitamin D deficiency population in MENA (population), intervention with vitamin D (intervention), control or placebo group (comparison), outcome parameters of the prediabetes and vitamin D level (outcome), and RCT studies (study). Two independent reviewers (MAMYAH and NR) conducted a detailed review of the titles, abstracts, and full-text articles, and any discrepancies were resolved through consultation with MAK and NAW. We included only RCTs on prediabetes that involved vitamin D supplements with patients aged 15 years and older, and conducted in the MENA region, and articles written in English. Further details regarding inclusion and exclusion criteria used for this systematic review are listed in Table 2.

### 2.3. Data Extraction and Analysis

Key data were meticulously extracted from each study, focusing on population characteristics, sample sizes, intervention details, outcomes, and risk of bias. This was done using a standardized data extraction form to ensure consistency and accuracy. The extracted data were synthesized using a narrative approach due to the heterogeneity among the studies in terms of intervention dosages, durations, and baseline vitamin D levels. A qualitative synthesis was conducted to summarize the findings across the studies. Descriptive statistics were used to summarize the study characteristics and outcomes. This comprehensive synthesis approach allowed for a nuanced understanding of the impact of vitamin D supplementation on prediabetic individuals in the MENA region.

### 2.4. Quality Assessment

MAMYAH and NR carried out an independent risk of bias assessment using the Cochrane ROB2 tool to ensure the quality of the articles included in the study, and any disagreements were further discussed with MAK and NAW [28]. We evaluated seven domains: random sequence generation, allocation concealment, blinding of participants and personnel, blinding of outcome assessors, incomplete outcome data, and other biases. Each domain was rated as low, unclear, or high risk. Studies were classified as low risk overall if all domains were low risk; they were considered high risk if any domain was unclear or high risk. Information was obtained for missing data on risk estimates through study protocols, direct communication with authors, or previous systematic reviews. We further illustrated the data by plotting a bar graph where we could see how many articles fulfilled each of the domains. In addition, the quality of evidence on selected articles was also assessed using Grades of Recommendation, Assessment, Development, and Evaluation (GRADE) [29].

## 3. Results

### 3.1. Study Selection

**Figure 4:**
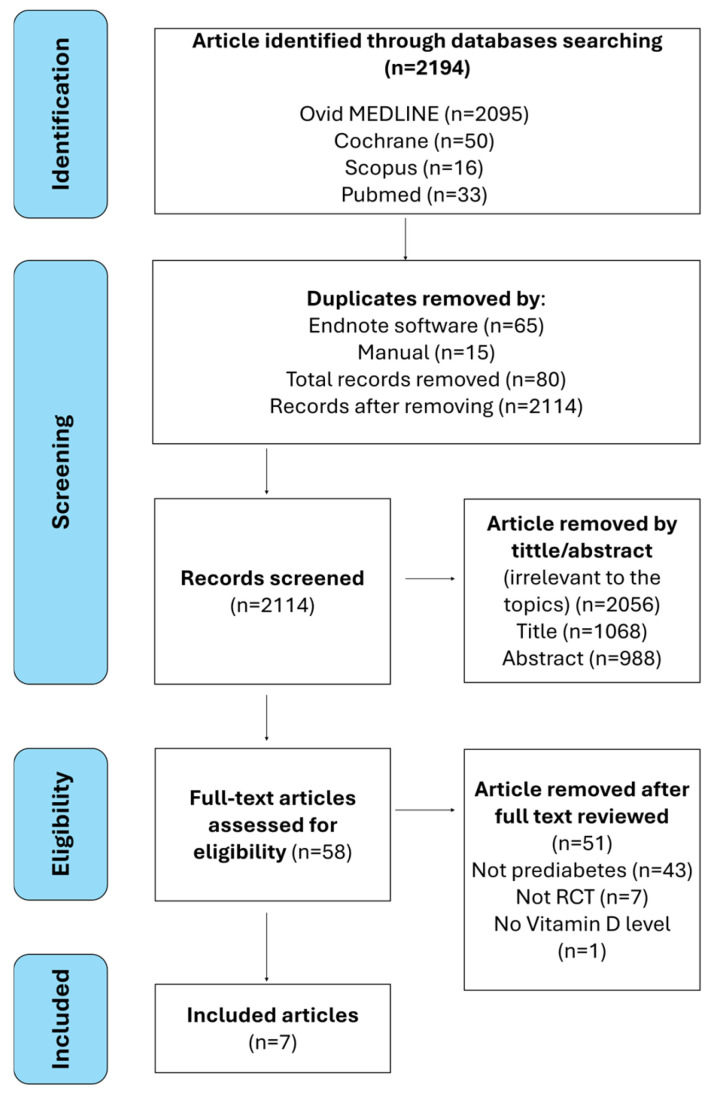
PRISMA flowchart of the process of the literature search and screening.

A total of 2194 studies were identified through database searches: MEDLINE (Ovid) (2095 studies), PubMed (33 studies), SCOPUS (16 studies), and Cochrane Central Register of Controlled Trials (CENTRAL) (50 studies). After removing 80 duplicates, 2114 studies remained for title and abstract screening. During the full-text screening, all records that did not fulfill the criteria were removed (Table 2). Most records were abstracts and proceedings, not studying prediabetes, non-RCT studies, and not involving vitamin D intervention, as shown in Figure 4. Ultimately, seven studies from the Middle East and North Africa (MENA) region met the inclusion criteria.

### 3.2. Description of the Studies

A total of seven studies were included in this systematic review, as shown in Table 3, conducted across various countries within the MENA region, including Iran, Qatar, Saudi Arabia, and Egypt [30,31,32,33,34,35,36]. The study population comprised prediabetic adults, and the sample sizes ranged from 42 to 203 participants. The mean age of participants varied from 36 to 48.1 years, and the proportion of female participants ranged from 17.5% to 100%. The studies employed different interventions, primarily involving vitamin D supplementation, with dosages ranging from 1000 IU/day to 50,000 IU per week, and the duration of interventions ranged from 2 to 6 months, as shown in Table 4.

**Figure 5:**
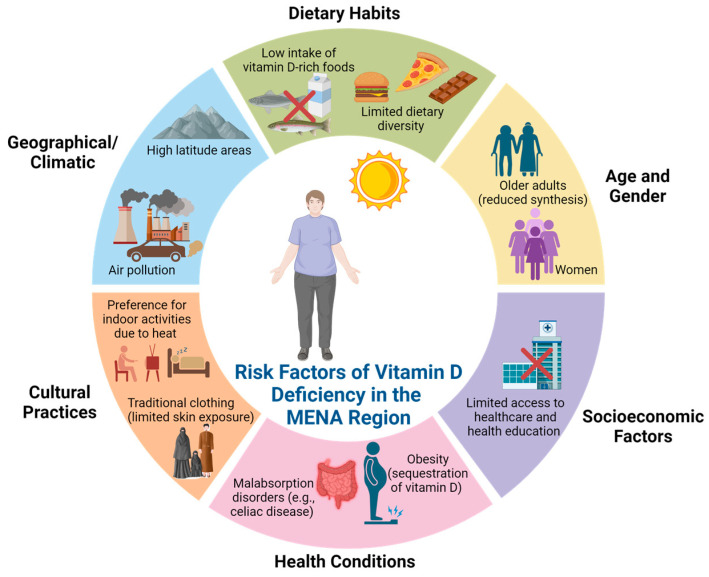
Risk factors of vitamin D deficiency in the MENA region. Created with BioRender.com, accessed on 2 August 2024.

The primary outcomes assessed across the studies included changes in blood glucose levels, insulin resistance, and the incidence of prediabetes. Most studies reported significant improvements in these parameters with vitamin D supplementation, indicating a beneficial effect on glycemic control and insulin sensitivity in prediabetic individuals, as shown in Table 4. Significant heterogeneity was observed among the studies in terms of intervention dosages, durations, and baseline vitamin D levels. A range of risk factors for vitamin D deficiency were identified in the included studies, as shown in Figure 5. The factors can be categorized into geographical or climatic factors, cultural practices, dietary habits, health conditions, age, gender, and socioeconomic factors (Figure 5). The detailed tables provide a comprehensive overview of the study characteristics and intervention outcomes, offering clear summaries of the data for easy interpretation.

### 3.3. Assessment of the Risk of Bias (RoB) Tool for RCTs and GRADE

**Figure 6:**
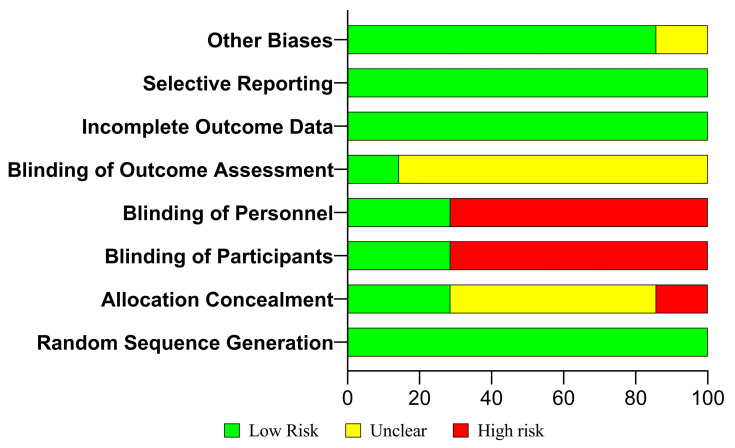
Risk of bias assessment analysis on the selected studies using Cochrane ROB tool.

Since this systematic review was conducted on RCTs, which are clinical studies, Cochrane ROB2 was used to assess the risk of bias in the selected studies. All included studies presented their data completely without any selective reporting, as shown in Table 5. All the studies also mentioned random sequence generation and incomplete outcome data (100%, *n* = 7). Most of the studies did not mention information on blinding of outcome assessment (85.71%, *n* = 6), except for one study conducted by Niroomand et al. (14.29%, *n* = 1). Blinding of personnel, blinding of participants, and allocation concealment received 28.47% from the assessment, and details can be seen in Figure 6. In summary, two of the studies had low risk while the other five studies had unclear risk.

The quality of evidence from selected studies was further evaluated using the Grades of Recommendation, Assessment, Development, and Evaluation (GRADE) and the assessment results are tabulated in Table 6. Niroomand et al. show a moderate positive effect with high-quality evidence due to low risk of bias, low inconsistency, and a large sample size with consistent outcomes [32]. Zarrin et al. report a moderate positive effect with high-quality evidence, supported by consistent outcomes despite moderate risk of bias [36]. Rajabi-Naeeni et al. show a positive effect with moderate-quality evidence, though the small sample size and co-supplementation introduce imprecision [33]. Rashad et al. report a moderate positive effect with low-quality evidence due to high risk of bias, high inconsistency, and a small sample size [35]. On the other hand, Al Thani et al. finds no significant effect, with moderate-quality evidence affected by moderate risk of bias and inconsistency, though the sample size was adequate. Ansari also finds no significant effect, with moderate-quality evidence affected by mixed outcomes and moderate risk of bias [31]. Finally, Hoseini et al. find no significant effect, with low-quality evidence due to high risk of bias, high inconsistency, and a small sample, making the results less reliable [34]. From all the studies, it is shown that larger sample sizes and consistent outcomes tend to yield higher-quality evidence, while smaller, inconsistent studies tend to produce weaker evidence.

## 4. Discussion

This systematic review examined seven studies that investigated the potential benefits of vitamin D supplementation in individuals with prediabetes characterized by impaired glucose tolerance and fasting glucose, serving as a precursor to type 2 diabetes. The studies assessed various outcomes, including glycemic control [30,31,32,33,34,35,36], insulin resistance [30,31,32,34,36], oxidative stress [30], and cardiometabolic risk factors [33,35]. A key strength of this review is the inclusion of randomized controlled trials (RCTs), which are considered the gold standard for evaluating the efficacy of interventions [30,31,32,33,34,35,36]. The diverse study populations included participants from Qatar [31], Iran [32,33,34,36], and Egypt [35], elderly individuals [31], reproductive-aged women [33], and obese individuals [35], providing valuable insights into the effects of vitamin D supplementation across different demographic and cultural contexts. However, it is important to consider the heterogeneity among the studies. They varied in terms of dosage and duration of vitamin D supplementation, baseline vitamin D status, and specific outcome measures assessed [30,31,32,33,34,35,36].

In this systematic review, we also performed ROB2 for the risk of bias assessment, which is specific to RCT studies, together with GRADE to evaluate the methodological quality and strength of evidence of the selected studies [37,38]. Our findings showed that most of the selected studies had 100% compliance in domain-selective reporting, incomplete outcome data, and random sequence generation, with lesser compliance in the domain of blinding of participants, personal assessment, and outcome assessment. By highlighting this shortcoming, future studies should take consideration of reporting details on these domains to improve their RCT bias assessment. This can also provide information on the current research gap in this topic, specifically in the MENA regions. By performing these ROB2 and GRADE analyses, we can also provide more confidence in presenting the roles of vitamin D supplementation in ameliorating the insulin sensitivity and metabolic health in people with prediabetes. This review carefully examines these differences and explores potential sources of heterogeneity, which may affect the interpretation and generalizability of the findings. The clinical significance of the reported effects is also evaluated. While statistically significant changes in outcome measures were observed, it is crucial to evaluate whether these findings lead to meaningful health improvements for individuals with prediabetes [30,31,32,33,34,35,36].

### 4.1. Potential Mechanisms of Vitamin D Influencing Glucose Metabolism and Type 2 Diabetes

The role of vitamin D supplementation in glycemic control, insulin resistance, and cardiometabolic health has been widely explored in recent research [30,31,32,33,34,35,36]. While vitamin D was traditionally associated with bone metabolism, emerging evidence highlights its involvement in glucose homeostasis and insulin sensitivity. A study by Rashad et al. specifically examined its effects on insulin resistance in prediabetic obese patients, demonstrating its potential to influence insulin sensitivity and glucose regulation [35]. However, factors such as obesity and insulin resistance may alter vitamin D metabolism and its overall impact, necessitating careful consideration when interpreting findings.

**Figure 7:**
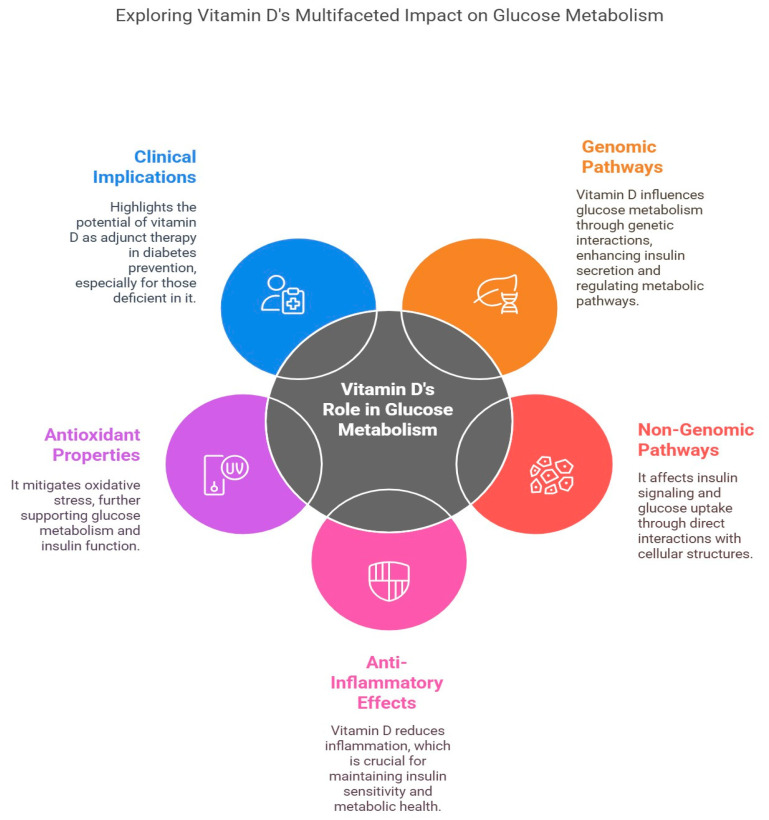
Depicts how vitamin D maintains normal blood sugar levels through several different ways in the human body. Vitamin D boosts metabolic function both through genes by making insulin efficient, and through cells by targeting how insulin operates and how glucose enters. The anti-inflammatory nature helps improve insulin utilization and metabolism, while its antioxidant action reduces oxidative stress to strengthen glucose control. Vitamin D has shown potential to help prevent diabetes when used alongside other treatments, especially for people who do not get enough vitamin D.

Mechanistically demonstrated in Figure 7, vitamin D regulates glucose metabolism through both genomic and non-genomic pathways. It enhances insulin secretion via calcium-dependent endopeptidases, modulates the PI3K/AKT signaling pathway, interacts with vitamin D receptor (VDR) and vitamin D-binding protein (DBP) gene variations, and reduces inflammation and secondary hyperparathyroidism, which are the key contributors to type 2 diabetes progression [39,40,41]. A study conducted on knockout VDR mice showed impairment in insulin resistance, and supplementation with vitamin D did improve insulin secretion by stimulating the pancreatic beta cells [42]. It was also found that vitamin D activates the VDR by forming a heterodimer with retinoid X receptor (RXR), which then binds to the vitamin D response elements (VDREs) in the nucleus of major metabolic tissues. This binding further induces changes in the level of gene and proteomic expression, which, in combination, lead to an improvement in the islet function and decrease insulin resistance [43]. Vitamin D enhances pancreatic beta-cell function by regulating insulin secretion and protecting against inflammation, oxidative stress, and apoptosis. This helps preserve beta-cell mass and insulin production, contributing to improved glucose homeostasis [44]. In particular, vitamin D receptors in insulin-sensitive tissues, such as muscle and fat, facilitate glucose uptake and utilization, with some studies reporting enhanced insulin signaling in muscle tissue, where most postprandial glucose uptake occurs [45]. Vitamin D also upregulates key insulin signal transduction enhancement elements, including PI3K/Akt and IRS-1 pathways, and promotes GLUT-4 expression and localization, thereby improving glucose uptake [46].

Inflammation and oxidative stress are critical factors in prediabetes, disrupting insulin signaling and promoting metabolic dysfunction. Vitamin D’s anti-inflammatory properties help mitigate these effects by reducing pro-inflammatory cytokines and supporting a more favorable inflammatory profile. Additionally, its antioxidant properties may counteract oxidative stress, which is often elevated in prediabetic individuals [45,47]. As vitamin D normalizes the redox state, it also mitigates oxidative stress-induced insulin resistance, and activates SIRT-1/AMPK pathways, thus improving glucose metabolism [48,49]. Despite these promising mechanisms, findings from meta-analyses of randomized controlled trials remain inconsistent. Variability in study populations, supplementation doses, and intervention durations may explain the discrepancies in observed effects. While some studies report improvements in insulin sensitivity and glucose regulation, others show minimal to no impact. However, individuals with low baseline vitamin D levels or those at higher risk for diabetes appear to benefit the most from supplementation [45,50]. Although vitamin D supplementation alone is not a substitute for lifestyle or pharmacological interventions, it is increasingly recognized as a beneficial adjunct therapy for prediabetes, particularly in individuals with vitamin D deficiency. Further high-quality research is needed to clarify its optimal dosing and long-term effects in metabolic disease prevention.

### 4.2. Risk Factors for Vitamin D Deficiency and Its Association with Type 2 Diabetes

The prevalence of vitamin D deficiency in the MENA region affecting children and adults ranged from 12% to 96% and 44% to 96%, respectively [11]. Contributing factors include limited sun exposure due to cultural practices, dietary insufficiencies, and genetic predisposition. The risk factors for individuals with prediabetes, such as obesity, sedentary lifestyles, and inadequate dietary intake of vitamin D-rich foods, were identified as shown in Figure 5. Geographical and climatic influences, such as living in high-latitude areas with limited sunlight, significantly impact vitamin D status [51]. Additionally, air pollution is increasingly recognized as a contributor to low vitamin D levels, as pollutants can reduce the amount of UVB radiation reaching the skin [52]. Cultural practices also play a role, for instance, traditional clothing that limits skin exposure, and a preference for indoor activities due to extreme heat can significantly reduce vitamin D synthesis [53,54].

Dietary habits play a crucial role in maintaining adequate vitamin D levels, and low intake of vitamin D-rich foods such as fish and fortified products, combined with limited nutritional diversity, can lead to deficiencies [55]. Certain health conditions like obesity, which causes the sequestration of vitamin D in fat tissue, and malabsorption disorders such as celiac disease, further increase the risk of deficiency [56]. Additionally, age and gender differences also affect vitamin D levels. Older adults typically have reduced capacity to produce vitamin D, while women are influenced by cultural and dietary factors [57]. Socioeconomic factors cannot be overlooked, as limited access to healthcare and health education significantly impact vitamin D status among women of childbearing age [58].

Consistent evidence from this review indicates a strong association between low vitamin D levels and prediabetes. Vitamin D deficiency is linked to impaired glucose metabolism, increased insulin resistance, and elevated fasting blood glucose levels, suggesting a critical role in the development of prediabetes [23,59]. However, some studies have found no significant association between vitamin D levels and prediabetes, suggesting that other factors might also play a crucial role in the pathogenesis of prediabetes [26]. These discrepancies highlight the need for further research to elucidate the complex relationship between vitamin D and prediabetes. Based on this systematic review, we found the impact of vitamin D supplementation on glycemic control in prediabetic individuals yielded mixed results. While some reported significant improvements in fasting blood glucose, HbA1c, and insulin sensitivity [31,32,33,35,36], others found no substantial effects on blood diabetic parameters like HOMA-IR and FSI [30,34]. These variations may be attributed to differences in study design, supplementation dosage, duration, and baseline vitamin D levels.

Future research on more rigorous, well-designed randomized controlled trials (RCTs) is necessary to establish the efficacy of vitamin D supplementation in preventing the progression from prediabetes to type 2 diabetes. Future research should also focus on standardizing dosages and durations of supplementation and identifying optimal 25-hydroxyvitamin D levels for glycemic control. Additionally, the role of genetic and environmental factors in modulating vitamin D response should be explored. Given the high prevalence of vitamin D deficiency and its potential link to prediabetes, public health strategies in the MENA region should prioritize the prevention and treatment of hypovitaminosis D. Initiatives could include increasing awareness about the importance of vitamin D, promoting dietary sources, and encouraging safe sun-exposure practices.

This study’s findings could have implications for tailored interventions to improve glycemic control and mitigate the progression to type 2 diabetes. Hoseini et al. (2013) examined the impact of oral vitamin D supplementation on insulin resistance in prediabetic patients. Over 12 weeks, the study involved 45 participants randomly assigned to receive either vitamin D or a placebo. Despite the intervention, the results indicated that vitamin D did not significantly influence insulin sensitivity during this period. Hence, they suggested conducting a longer-duration randomized double-blind study to assess the potential effects of vitamin D supplements on insulin resistance [34]. Finally, this review addresses the potential limitations of the included studies, such as small sample sizes, short-term follow-up periods, and possible confounding factors. These limitations are acknowledged, and this review recommends areas for future research to elucidate the role of vitamin D supplementation in managing prediabetes and preventing the development of type 2 diabetes.

### 4.3. Strengths and Limitations of the Included Studies

This systematic review has good homogeneity in the reviewed study designs since we only included RCTs. RCTs provide robust evidence due to their rigorous design, including randomization, blinding, and control groups. We also included diverse study populations within this systematic review, where, despite being RCTs, the studies involve diverse populations from different countries within the MENA region (Iran, Qatar, Saudi Arabia, and Egypt). This diversity enhances the generalizability of findings beyond a single population. We provided comprehensive outcome measures within RCTs, where this review covers a range of outcome markers related to prediabetes status that are significant during the study, including fasting plasma glucose (FPG), 2 h oral glucose tolerance test (OGTT), HOMA-IR, and HbA1c. This comprehensive approach ensures a thorough assessment of vitamin D supplementation effects within the RCT framework. Since we limited the review to only articles written in English, this systematic review ensures consistency in language interpretation. This approach facilitates communication and accessibility.

Small sample sizes were used in some RCTs; for example, Rashad [35] only had 42 participants, while Hoseini et al. [34] had 45 participants. This may limit the statistical power and precision of effect estimates. In addition, the variability in baseline vitamin D levels is also observed, where the included RCTs report different baseline vitamin D levels (ranging from 12.44 nmol/L to 35 ng/mL). This variability may impact the interpretation of treatment effects. The duration of RCTs in the included studies was also observed, such as Rajabi-Naeeni et al. [33], which used a short intervention duration (8 weeks). Longer follow-up periods would provide insights into sustained effects. Conflicting findings on glucose tolerance and insulin sensitivity among the selected studies can also be seen. For instance, studies such as Al Thani et al. [31] report contradictory results regarding glucose tolerance and insulin sensitivity.

### 4.4. Recommendations on Public Health Strategies in MENA Region

There is an abundance of studies that showed an association between vitamin D deficiency and diabetes progression, which was identified to affect several important metabolic pathways; thus, hypovitaminosis D has become a significant public health concern, specifically in the MENA region. As previously described, despite abundant sunshine, cultural practices, dark skin color, and hot climates limit sun exposure; the prevalence rates of this deficiency are high, and range from 30 to 90% [60,61]. Public health strategies to address this problem should include education on safe sun exposure, dietary recommendations, and supplementation programs [62]. Additionally, to effectively address this issue, food fortification policies and vitamin D supplementation are suggested as long-term strategies, with support and help from governments by recognizing vitamin D deficiency as a public health concern and allocating resources accordingly [63].

There are important implications of our findings for the development of more targeted paradigms in public health to counteract the growing concern of type 2 diabetes in the MENA region. This population requires selective initiatives based on their demographic, genetic, and environmental conditions, with reference to vitamin D in diabetic populations. The results presented here provide support for several central tenets of multiple public health interventions that could potentially greatly influence diabetes care and prevention strategies.

Firstly, vitamin D supplementation programs could be very effective in dealing with deficiency in the MENA region because many people of this region, including women and children, cover their bodies to avoid sun exposure due to cultural or personal preferences, and, as a result, people often suffer vitamin D deficiency. This analysis has demonstrated that vitamin D supplementation can enhance glucose metabolism and insulin sensitivity in persons with pre-diabetic states [64]. Vitamin D supplements could be made available at subsidized or even free rates within countries through public health programs; the target groups would include those with prediabetes.

However, supplementation alone should always be supported by dietary guidelines that encourage the intake of foods that contain vitamin D. Governments and other relevant agencies involved in public health crusades should encourage the use of foods rich in nutrients, such as dairy products, fatty fish, and egg yolks, to contain the scourge of dietary deficiencies. For instance, existing studies depict that food fortification programs have effectively addressed the problem of low levels of vitamin D in other areas [65]. Others could also focus on engendering correct eating habits by school and community organizations that include vitamin D sources.

However, safe sun exposure appears to be an essential matter to be focused on in order to avoid the dangerous health effects associated with skin cancer. Since most of the region receives plenty of sunlight, it is relevant to provide information on how to get the best out of the sun by ensuring adequate vitamin D production without causing skin harm. For example, exposure of the skin during appropriate hours of the day, which has a short duration but a high frequency, can help in the achievement of adequate vitamin D without escalation of skin cancer risks [66]. Such barriers include dressing codes or the belief that Black Caribbean skin is not affected by the sun.

Incorporating the screening and monitoring of vitamin D levels in high-risk clients into the health setting should be done. A combination of assessment of vitamin D status at least annually and determining its frequency based on the patients’ risk factors, like prediabetes and metabolic syndrome, appears optimal. This review also emphasized the need to provide healthcare providers with knowledge of identifying and preventing vitamin D deficiency as part of the broader strategy of containing diabetes.

Last, but not least, it is critical to become familiar with regional research funding. As our study points out, research with better study designs, higher sample completion, longer follow-up, and uniform baseline serum vitamin D levels is important for determining a cause–effect relationship between vitamin D and diabetes. The above evidence can help the policymakers set up clinical-care guidelines relevant to the region. Thirdly, such research will be equipped with specific funding to guarantee that future efforts in public health are founded on valid and contextual knowledge.

Therefore, the listed strategies aligned to our discoveries stress the evidence-based approaches to deal with the increasing burden of diabetes in the MENA region. With supplementation, education, diets, and disease prevention, public concerns will go far in minimizing the effects of type 2 diabetes on the region’s population.

## 5. Conclusions

This systematic review highlights the impact of vitamin D supplementation on prediabetes across various populations within the MENA region. The included randomized controlled trials (RCTs) demonstrate the potential of high-dose vitamin D to improve insulin sensitivity and reduce the progression to diabetes in prediabetic individuals, as evidenced by studies such as Niroomand et al. [32] and Zarrin [36]. However, the findings are not unanimous, with some studies such as Al Thani et al. [31] reporting no significant effect on glucose tolerance or insulin sensitivity.

The strengths of this review lie in its focus on RCTs, which provide robust evidence, and the inclusion of diverse study populations, enhancing the generalizability of the findings. Comprehensive outcome measures, including FPG, OGTT, and HbA1c, offer a thorough assessment of the effects of vitamin D supplementation. However, limitations such as small sample sizes, variability in baseline vitamin D levels, and short intervention durations must be considered. Additionally, the conflicting findings on glucose tolerance and insulin sensitivity highlight the need for further research to clarify these discrepancies.

These results show how important it is to conduct well-designed studies that focus on a specific area to identify the role of vitamin D in preventing type 2 diabetes, especially in the MENA region, which has its own unique population and environment. This kind of study could help shape public health plans and clinical guidelines to deal with the rising number of people in this group who have diabetes. Future studies should aim for larger sample sizes, longer follow-up periods, and consistent baseline vitamin D levels to provide more definitive evidence on the role of vitamin D in managing prediabetes. Addressing these limitations will contribute to more precise and actionable recommendations for clinical practice and public health policies.

## Figures and Tables

**Table 1:** Search strategies for different databases were used for the literature search.

Database	Search Strategy
Ovid MEDLINE	((“prediabetes” or “impaired glucose tolerance” or “IGT” or “insulin resistance”) and (“Vitamin D” or “25-hydroxyvitamin D” or “cholecalciferol”) and (“oral glucose tolerance test” or “OGTT” or “fasting blood sugar” or “fbs” or “fasting blood glucose” or “fbg” or “hba1c”) and (“north africa *” or “morroco” or “algeria” or “tunisi *” or “libya” or “egypt” or “sudan” or “north africa” or “middle east” or “arab *” or “saud *” or “iran” or “iraq” or “lebanon” or “kuwait” or “yemen” or “israel” or “jordan” or “oman” or “syria” or “qatar” or “bahrain” or “turk *” or “palesti *”) and (“randomized controlled trial” or “randomized controll trial” or “RCT”)).af.
Cochrane	“prediabetes” OR “impaired glucose tolerance” OR “IGT” OR “insulin resistance” in All Text AND “Vitamin D” OR “25-hydroxyvitamin D” OR “cholecalciferol” in All Text AND “oral glucose tolerance test” OR “OGTT” OR “fasting blood sugar” OR “fbs” OR “fasting blood glucose” OR “fbg” OR “hba1c” in All Text AND “north africa *” OR “morroco” OR “algeria” OR “tunisi *” OR “libya” OR “egypt” OR “sudan” OR “north africa” OR “middle east” OR “arab *” OR “saud *” OR “iran” OR “iraq” OR “lebanon” OR “kuwait” OR “yemen” OR “israel” OR “jordan” OR “oman” OR “syria” OR “qatar” OR “bahrain” OR “turk *” OR “palesti *” in All Text AND “randomized controlled clinical trial” OR “randomized controlled trial” OR “RCT” in All Text
Scopus	(TITLE-ABS-KEY (“prediabetes” OR “impaired glucose tolerance” OR “IGT” OR “insulin resistance”) AND TITLE-ABS-KEY (“Vitamin D” OR “25-hydroxyvitamin D” OR “cholecalciferol”) AND TITLE-ABS-KEY (“oral glucose tolerance test” OR “OGTT” OR “fasting blood sugar” OR “fbs” OR “fasting blood glucose” OR “fbg” OR “hba1c”) AND TITLE-ABS-KEY (“north africa *” OR “morroco” OR “algeria” OR “tunisi *” OR “libya” OR “egypt” OR “sudan” OR “north africa” OR “middle east” OR “arab *” OR “saud *” OR “iran” OR “iraq” OR “lebanon” OR “kuwait” OR “yemen” OR “israel” OR “jordan” OR “oman” OR “syria” OR “qatar” OR “bahrain” OR “turk *” OR “palesti *”) AND TITLE-ABS-KEY (“randomized controlled trial” OR “randomized controll trial” OR “RCT”))
PubMed	((((“prediabetes” OR “impaired glucose tolerance” OR “IGT” OR “insulin resistance”) AND (“Vitamin D” OR “25-hydroxyvitamin D” OR “cholecalciferol”)) AND (“oral glucose tolerance test” OR “OGTT” OR “fasting blood sugar” OR “fbs” OR “fasting blood glucose” OR “fbg” OR “hba1c”)) AND (“north africa *” OR “morroco” OR “algeria” OR “tunisi” OR “libya” OR “egypt” OR “sudan” OR “north africa” OR “middle east” OR “arab *” OR “saud *” OR “iran” OR “iraq” OR “lebanon” OR “kuwait” OR “yemen” OR “israel” OR “jordan” OR “oman” OR “syria” OR “qatar” OR “bahrain” OR “turk *” OR “palesti *”)) AND (“randomized controlled trial” OR “randomized controll trial” OR “RCT”)

Abbreviation used depends on the database: ABS: Abstract; KEY: Keywords; *: wildcard symbol

**Table 2:** Selection and exclusion criteria for literature screening.

Inclusion	Exclusion
Adults (≥15 years old) with prediabetes living in the MENA (Middle East and North Africa) region.	Children and adolescents (<15 years old). Individuals without prediabetes. Individuals living outside the MENA region.
Studies evaluating vitamin D supplementation or assessing vitamin D (25-hydroxyvitamin D) status.	Studies that do not evaluate vitamin D supplementation or vitamin D status.
Measures of prediabetes status (e.g., fasting plasma glucose, 2-h oral glucose tolerance test, HbA1c)	Studies that do not report outcomes related to prediabetes status or progression to type 2 diabetes.
Randomized controlled trials (RCTs).	Cross-sectional studies (CSSs), case–control studies (CCSs), systematic reviews or meta-analyses, case reports, case series, editorials, letters, commentaries, and conference abstracts.
Studies published in the English language.	Studies published in languages other than English.

**Table 3:** Summaries of the included studies, patient demographic data and intervention.

Author, Year	Country	No of Patients	Mean Age (Years)	Mean BMI (kg/m^2^)	% Female	Duration	Control	Vitamin D Dose (IU)
Ansari 2020 [30]	KSA	203	Saudi Adult 30–60.	NAD	61.57	6 months	placebo	Vit D3 50,000 IU/w for 2 months, 50,000 IU every other week for 2 months 1000 IU/d for 2 months
Al Thani 2019 [31]	Qatar	132	45.51	30.0/32.0	17.5	6 months	Placebo	Vit D3 30,000 IU/w
Niroomand 2019 [32]	Iran	162	46.5	31.5	76.5	6 months	Placebo	Vit D3 50,000 IU/w for 3 months, then 50,000 IU/month
Rajabi-Naeeni 2020 [33]	Iran	168	Reproductive age	27.28	100	8 weeks	Placebo	Vit D3 50,000 IU/w
Hoseini 2013 [34]	Iran	45	47.4	29.4	71.1	3 months	Vitamin D IM + ca carbonate	Vit D supplement 50,000 IU/w
Rashad 2023 [35]	Egypt	42	36	35.143	90.47	3 months	Weight-reduction diet only	Vit D3 50,000 IU/w for 2 months followed by 25,000 IU for one month
Zarrin 2017 [36]	Iran	104	48.1	28.71 ±4.29	51.9	3 months	placebo	Vit D3 1000 IU/d

Abbreviations used: BMI: body mass index; IM: intramuscular; NAD: not available; Vit D3: Vitamin D.

**Table 4:** Level of vitamin D at the baseline and post-intervention and its effect on the outcome markers.

Author, Year	Title	Outcome Markers	Baseline 25 OH D for Vitamin D/Control Groups (nmol/L)	Achieved 25 OH D for Vitamin D/Control Groups (nmol/L)	Result (Effect of Vitamin D Supplementation on Prediabetes)
Ansari 2020 [30]	Vitamin D Supplementation is Associated with Increased Glutathione Peroxidase-1 Levels in Arab Adults with Prediabetes	GPx1 ↑, Vit D3 ↑	32.5/31.9	66.2/29.10	Vitamin D supplementation modulates GPx1 levels that can favorably benefit vitamin D-deficient patients with prediabetes,
Al Thani 2019 [31]	The effect of vitamin D supplementation on the glycemic control of pre-diabetic Qatari patients in a randomized control trial	Weight ↓, BMI ↓, FBS ↓, FSI ↓, HOMA-IR ↓, Vit D3 ↑	14.9/17.0 ng/mL	34.3/16.1 ng/mL	No effect on glucose tolerance or insulin sensitivity
Niroomand 2019 [32]	Does high-dose vitamin D supplementation impact insulin resistance and risk of development of diabetes in patients with pre-diabetes? A double-blind randomized clinical trial	Vit D3 ↑ FSI ↓ HOMA-IR ↓	13.7/13.7	36.0/16.0	High-dose vitamin D supplementation improved insulin sensitivity and reduced the risk of progression to diabetes in patients with prediabetes.
Rajabi-Naeeni 2020 [33]	The effect of co supplementation of omega-3 and vitamin D on cardio metabolic risk factors and psychological distress in reproductive-aged women with prediabetes and hypovitaminosis D: a study protocol for a randomized controlled trial	Weight ↓, FBS ↓, FBI ↓, HOMA-IR ↓, HOMA-β ↓, HDL ↑, LDL ↓, Vit D3 ↑	21.43/25.47	29.71/20.97	Vitamin D and omega-3 co-supplementation improved glycemic control, HDL-cholesterol levels, and weight in women with prediabetes and hypovitaminosis D.
Hoseini 2013 [34]	The effects of oral vitamin D on insulin resistance in pre-diabetic patients	Vit D3 ↑	77.5/80 77.5/44.8	118.8/102.8 118.8/34.6	Oral vitamin D did not affect insulin sensitivity in pre-diabetes patients in 12 weeks of treatment.
Rashad 2023 [35]	Vitamin D Supplementation Influence in Insulin-Resistant Pre-Diabetic Obese Patients.	DBP ↓, HOMA-β ↓, Vit D3 ↑, PTH ↓, ALP ↓ Bilirubin ↓	12.69/12.44	30.93/24.49	Increasing vitamin D status in pre-diabetic, insulin-resistant obese individuals with vitamin D deficiency were associated with reduced weight and improved insulin resistance.
Zarrin 2017 [36]	The Effect of Vitamin D Supplementation on the Glycemic Status and the Percentage of Body Fat Mass in Adults with Prediabetes: A Randomized Clinical Trial	FBS ↓, Vit D3 ↑	19.36/24.16	30.48/22.29	The study demonstrated that 1000 IU vitamin D supplementation for 3 months decreased insulin resistance in individuals with prediabetes.

Abbreviations used: ALP: alkaline phosphatase; BMI: body mass index; FBS: fasting blood sugar; FBI: fasting blood insulin; FSI: fasting serum insulin; GPx1: Glutathione peroxidase 1; HOMA-β: homeostasis model assessment of β-cell function; HOMA-IR: homeostasis model assessment for insulin resistance; HDL: high-density lipoprotein; LDL: low-density lipoprotein; PTH: parathyroid hormone; Vit D3: Vitamin D; ↑: increase; ↓: decrease.

**Table 5:** Risk of bias assessment on the selected studies using Cochrane ROB tool.

Study	Random Sequence Generation	Allocation Concealment	Blinding of Participants	Blinding of Personnel	Blinding of Outcome Assessment	Incomplete Outcome Data	Selective Reporting	Other Biases
Ansari (2020) [30]	🟢 Low Risk	🔴 High Risk	🔴 High Risk	🔴 High Risk	🟡 Unclear	🟢 Low Risk	🟢 Low Risk	🟢 Low Risk
Al Thani (2019) [31]	🟢 Low Risk	🟡 Unclear	🔴 High Risk	🔴 High Risk	🟡 Unclear	🟢 Low Risk	🟢 Low Risk	🟢 Low Risk
Niroomand (2019) [32]	🟢 Low Risk	🟢 Low Risk	🟢 Low Risk	🟢 Low Risk	🟢 Low Risk	🟢 Low Risk	🟢 Low Risk	🟡 Unclear
Rajabi-Naeeni (2020) [33]	🟢 Low Risk	🟢 Low Risk	🟢 Low Risk	🟢 Low Risk	🟡 Unclear	🟢 Low Risk	🟢 Low Risk	🟢 Low Risk
Hoseini (2013) [34]	🟢 Low Risk	🟡 Unclear	🔴 High Risk	🔴 High Risk	🟡 Unclear	🟢 Low Risk	🟢 Low Risk	🟢 Low Risk
Rashad (2023) [35]	🟢 Low Risk	🟡 Unclear	🔴 High Risk	🔴 High Risk	🟡 Unclear	🟢 Low Risk	🟢 Low Risk	🟢 Low Risk
Zarrin (2017) [36]	🟢 Low Risk	🟡 Unclear	🔴 High Risk	🔴 High Risk	🟡 Unclear	🟢 Low Risk	🟢 Low Risk	🟢 Low Risk

🟢 Low Risk 🟡 Unclear 🔴 High Risk.

**Table 6:** Quality of evidence on selected articles using Grades of Recommendation, Assessment, Development, and Evaluation (GRADE).

Studies	Effect	Risk of Bias	Inconsistency	Indirectness	Imprecision	Quality of Evidence	Other Considerations
Ansari (2020) [30]	No significant effect	Moderate	Moderate	None	Moderate	Moderate	Significant increase in GPx1, mixed
Al Thani (2019) [31]	No significant effect	Moderate	Moderate	None	Moderate	Moderate	Mixed outcomes, adequate sample
Niroomand (2019) [32]	Moderate positive	Low	Low	None	Low	High	Large sample size, consistent outcomes
Rajabi-Naeeni (2019) [33]	Positive	Low	Low	None	Moderate	Moderate	Co-supplementation
Hoseini (2013) [34]	No significant effect	Moderate	High	None	High	Low	Small sample size
Rashad (2023) [35]	Moderate positive	Moderate	High	None	High	Low	Small sample size
Zarrin (2017) [36]	Moderate positive	Moderate	Low	None	Low	High	Consistent positive outcomes

## Data Availability

Data for the screening and other datasets used in this study are available on request from corresponding authors.

## Supplementary Materials

The following supporting information can be downloaded at: https://www.mdpi.com/article/10.3390/jcm14041239/s1, Tables S1–S4: Search string strategy; Table S1: Cochrane search strategy; Table S2: Scopus search strategy; Table S3: Ovid MEDLINE search strategy; Table S4: Pubmed search strategy.
