# Supplementary material for: Evaluating the Role of Vitamin D in Prediabetes Management, Insights from RCTs in the MENA Region: A Comprehensive Systematic Review
Source: J Clin Med. 2025 Feb 13;14(4):1239. doi: 10.3390/jcm14041239 (PMC11856371; doi:10.3390/jcm14041239)
Supplement: Supplementary file 1 [file jcm-14-01239-s001.zip › jcm-3376213-supplementary.pdf]

SEARCH STRATEGY

Search will be done in Cochrane, Scopus and Ovid MEDLINE database on 9-12 September 2024 using MESH term as below:

Population	"prediabetes" OR "impaired glucose tolerance" OR "IGT" OR "insulin resistance"
Intervention	"Vitamin D" OR "25-hydroxyvitamin D" OR "cholecalciferol"
Outcomes	"oral glucose tolerance test" OR "OGTT" OR "fasting blood sugar" OR “fbs” OR "fasting blood glucose" OR “fbg” OR "hba1c"
Population	"north africa*" OR "morroco" OR "algeria" OR "tunisi*" OR "libya" OR "egypt" OR "sudan" OR "north africa" OR “middle east” OR "arab*" OR "saud*" OR "iran" OR "iraq" OR "lebanon" OR "kuwait" OR "yemen" OR "israel" OR "jordan" OR "oman" OR "syria" OR "qatar" OR "bahrain" OR "turk*" OR "palesti*"
Study	"randomized controlled trial" OR "randomized controll trial" OR "RCT"

Table S1: Cochrane search strategy

Search ID	SEARCH	Item Found
#1	"prediabetes" OR "impaired glucose tolerance" OR "IGT" OR "insulin resistance" in All Text	22605
#2	"Vitamin D" OR "25-hydroxyvitamin D" OR "cholecalciferol" in All Text	16418
#3	"oral glucose tolerance test" OR "OGTT" OR "fasting blood sugar" OR “fbs” OR "fasting blood glucose" OR “fbg” OR "hba1c" in All Text	37260
#4	"prediabetes" OR "impaired glucose tolerance" OR "IGT" OR "insulin resistance" in All Text AND "Vitamin D" OR "25-hydroxyvitamin D" OR "cholecalciferol" in All Text AND "oral glucose tolerance test" OR "OGTT" OR "fasting blood sugar" OR “fbs” OR "fasting blood glucose" OR “fbg” OR "hba1c" in All Text	329
#5	"prediabetes" OR "impaired glucose tolerance" OR "IGT" OR "insulin resistance" in All Text AND "Vitamin D" OR "25-hydroxyvitamin D" OR "cholecalciferol" in All Text AND "oral glucose tolerance test" OR "OGTT" OR "fasting blood sugar" OR “fbs” OR "fasting blood glucose" OR “fbg” OR "hba1c" in All Text AND "north africa*" OR "morroco" OR "algeria" OR "tunisi*" OR "libya" OR "egypt" OR "sudan" OR "north africa" OR “middle east” OR "arab*" OR "saud*" OR "iran" OR "iraq" OR "lebanon" OR "kuwait" OR "yemen" OR "israel" OR "jordan" OR "oman" OR "syria" OR "qatar" OR "bahrain" OR "turk*" OR "palesti*" in All Text	60
#6	"prediabetes" OR "impaired glucose tolerance" OR "IGT" OR "insulin resistance" in All Text AND "Vitamin D" OR "25-	45

	hydroxyvitamin D" OR "cholecalciferol" in All Text AND "oral glucose tolerance test" OR "OGTT" OR "fasting blood sugar" OR "fbs" OR "fasting blood glucose" OR "fbg" OR "hba1c" in All Text AND "north africa*" OR "morroco" OR "algeria" OR "tunisi*" OR "libya" OR "egypt" OR "sudan" OR "north africa" OR "middle east" OR "arab*" OR "saud*" OR "iran" OR "iraq" OR "lebanon" OR "kuwait" OR "yemen" OR "israel" OR "jordan" OR "oman" OR "syria" OR "qatar" OR "bahrain" OR "turk*" OR "palesti*" in All Text AND "randomized controlled clinical trial" OR "randomized controlled trial" OR "RCT" in All Text	

Table S2: Scopus search strategy.

Search ID	SEARCH	Item Found
#1	TITLE-ABS-KEY ("prediabetes" OR "impaired glucose tolerance" OR "IGT" OR "insulin resistance")	202936
#2	TITLE-ABS-KEY ("Vitamin D" OR "25-hydroxyvitamin D" OR "cholecalciferol")	159394
#3	TITLE-ABS-KEY ("oral glucose tolerance test" OR "OGTT" OR "fasting blood sugar" OR "fbs" OR "fasting blood glucose" OR "fbg" OR "hba1c")	159234
#4	(TITLE-ABS-KEY ("prediabetes" OR "impaired glucose tolerance" OR "IGT" OR "insulin resistance") AND TITLE-ABS-KEY ("Vitamin D" OR "25-hydroxyvitamin D" OR "cholecalciferol") AND TITLE-ABS-KEY ("oral glucose tolerance test" OR "OGTT" OR "fasting blood sugar" OR "fbs" OR "fasting blood glucose" OR "fbg" OR "hba1c"))	847
#5	(TITLE-ABS-KEY ("prediabetes" OR "impaired glucose tolerance" OR "IGT" OR "insulin resistance") AND TITLE-ABS-KEY ("Vitamin D" OR "25-hydroxyvitamin D" OR "cholecalciferol") AND TITLE-ABS-KEY ("oral glucose tolerance test" OR "OGTT" OR "fasting blood sugar" OR "fbs" OR "fasting blood glucose" OR "fbg" OR "hba1c") AND TITLE-ABS-KEY ("north africa*" OR "morroco" OR "algeria" OR "tunisi*" OR "libya" OR "egypt" OR "sudan" OR "north africa" OR "middle east" OR "arab*" OR "saud*" OR "iran" OR "iraq" OR "lebanon" OR "kuwait" OR "yemen" OR "israel" OR "jordan" OR "oman" OR "syria" OR "qatar" OR "bahrain" OR "turk*" OR "palesti*"))	86
#6	(TITLE-ABS-KEY ("prediabetes" OR "impaired glucose tolerance" OR "IGT" OR "insulin resistance") AND TITLE-ABS-KEY ("Vitamin D" OR "25-hydroxyvitamin D" OR	16

	"cholecalciferol") AND TITLE-ABS-KEY ("oral glucose tolerance test" OR "OGTT" OR "fasting blood sugar" OR "fbs" OR "fasting blood glucose" OR "fbg" OR "hba1c") AND TITLE-ABS-KEY ("north africa*" OR "morroco" OR "algeria" OR "tunisi*" OR "libya" OR "egypt" OR "sudan" OR "north africa" OR "middle east" OR "arab*" OR "saud*" OR "iran" OR "iraq" OR "lebanon" OR "kuwait" OR "yemen" OR "israel" OR "jordan" OR "oman" OR "syria" OR "qatar" OR "bahrain" OR "turk*" OR "palesti*") AND TITLE-ABS-KEY ("randomized controlled trial" OR "randomized controll trial" OR "RCT"))	

Table S3: Ovid MEDLINE search strategy.

Search ID	SEARCH	Item Found
#1	"prediabetes" OR "impaired glucose tolerance" OR "IGT" OR "insulin resistance" {Including Limited Related Terms}	6336
#2	"Vitamin D" OR "25-hydroxyvitamin D" OR "cholecalciferol" {Including Limited Related Terms}	7765
#3	"oral glucose tolerance test" OR "fasting blood sugar" OR "fasting blood glucose" OR "hba1c" {Including Limited Related Terms}	5656
#4	(("prediabetes" or "impaired glucose tolerance" or "IGT" or "insulin resistance") and ("Vitamin D" or "25-hydroxyvitamin D" or "cholecalciferol") and ("oral glucose tolerance test" or "OGTT" or "fasting blood sugar" or "fbs" or "fasting blood glucose" or "fbg" or "hba1c")).af.	5055
#5	(("prediabetes" or "impaired glucose tolerance" or "IGT" or "insulin resistance") and ("Vitamin D" or "25-hydroxyvitamin D" or "cholecalciferol") and ("oral glucose tolerance test" or "OGTT" or "fasting blood sugar" or "fbs" or "fasting blood glucose" or "fbg" or "hba1c") and ("north africa*" or "morroco" or "algeria" or "tunisi*" or "libya" or "egypt" or "sudan" or "north africa" or "middle east" or "arab*" or "saud*" or "iran" or "iraq" or "lebanon" or "kuwait" or "yemen" or "israel" or "jordan" or "oman" or "syria" or "qatar" or "bahrain" or "turk*" or "palesti*")).af.	1931
#6	(("prediabetes" or "impaired glucose tolerance" or "IGT" or "insulin resistance") and ("Vitamin D" or "25-hydroxyvitamin D" or "cholecalciferol") and ("oral glucose tolerance test" or "OGTT" or "fasting blood sugar" or "fbs" or "fasting blood glucose" or "fbg" or "hba1c") and ("north africa*" or "morroco" or "algeria" or "tunisi*" or "libya" or "egypt" or "sudan" or "north africa" or "middle east" or "arab*" or "saud*" or "iran" or "iraq" or "lebanon" or "kuwait" or "yemen" or "israel" or "jordan" or "oman" or "syria" or "qatar" or "bahrain" or "turk*" or "palesti*") and ("randomized controlled trial" or "randomized controll trial" or "RCT")).af.	853

Table S4: Pubmed search strategy.

Search ID	SEARCH	Item Found
#1	"prediabetes" OR "impaired glucose tolerance" OR "IGT" OR "insulin resistance"	141834
#2	"Vitamin D" OR "25-hydroxyvitamin D" OR "cholecalciferol"	9738
#3	"oral glucose tolerance test" OR "OGTT" OR "fasting blood sugar" OR "fbs" OR "fasting blood glucose" OR "fbg" OR "hba1c"	10230
#4	(("prediabetes" OR "impaired glucose tolerance" OR "IGT" OR "insulin resistance") AND ("Vitamin D" OR "25-hydroxyvitamin D" OR "cholecalciferol")) AND ("oral glucose tolerance test" OR "OGTT" OR "fasting blood sugar" OR "fbs" OR "fasting blood glucose" OR "fbg" OR "hba1c")	48
#5	((("prediabetes" OR "impaired glucose tolerance" OR "IGT" OR "insulin resistance") AND ("Vitamin D" OR "25-hydroxyvitamin D" OR "cholecalciferol")) AND ("oral glucose tolerance test" OR "OGTT" OR "fasting blood sugar" OR "fbs" OR "fasting blood glucose" OR "fbg" OR "hba1c")) AND ("north africa*" OR "morroco" OR "algeria" OR "tunisi*" OR "libya" OR "egypt" OR "sudan" OR "north africa" OR "middle east" OR "arab*" OR "saud*" OR "iran" OR "iraq" OR "lebanon" OR "kuwait" OR "yemen" OR "israel" OR "jordan" OR "oman" OR "syria" OR "qatar" OR "bahrain" OR "turk*" OR "palesti*")	15
#6	(((("prediabetes" OR "impaired glucose tolerance" OR "IGT" OR "insulin resistance") AND ("Vitamin D" OR "25-hydroxyvitamin D" OR "cholecalciferol")) AND ("oral glucose tolerance test" OR "OGTT" OR "fasting blood sugar" OR "fbs" OR "fasting blood glucose" OR "fbg" OR "hba1c")) AND ("north africa*" OR "morroco" OR "algeria" OR "tunisi*" OR "libya" OR "egypt" OR "sudan" OR "north africa" OR "middle east" OR "arab*" OR "saud*" OR "iran" OR "iraq" OR "lebanon" OR "kuwait" OR "yemen" OR "israel" OR "jordan" OR "oman" OR "syria" OR "qatar" OR "bahrain" OR "turk*" OR "palesti*")) AND ("randomized controlled trial" OR "randomized controll trial" OR "RCT")	31
